# Creatine Kinase–Mediated ATP Supply Fuels Actin-Based Events in Phagocytosis 

**DOI:** 10.1371/journal.pbio.0060051

**Published:** 2008-03-11

**Authors:** Jan W. P Kuiper, Helma Pluk, Frank Oerlemans, Frank N van Leeuwen, Frank de Lange, Jack Fransen, Bé Wieringa

**Affiliations:** 1 Department of Cell Biology, Radboud University, Nijmegen Medical Centre, Nijmegen, The Netherlands; 2 Laboratory of Pediatric Oncology, Radboud University, Nijmegen Medical Centre, Nijmegen, The Netherlands; University of California–San Francisco, United States of America

## Abstract

Phagocytosis requires locally coordinated cytoskeletal rearrangements driven by actin polymerization and myosin motor activity. How this actomyosin dynamics is dependent upon systems that provide access to ATP at phagosome microdomains has not been determined. We analyzed the role of brain-type creatine kinase (CK-B), an enzyme involved in high-energy phosphoryl transfer. We demonstrate that endogenous CK-B in macrophages is mobilized from the cytosolic pool and coaccumulates with F-actin at nascent phagosomes. Live cell imaging with XFP-tagged CK-B and β-actin revealed the transient and specific nature of this partitioning process. Overexpression of a catalytic dead CK-B or CK-specific cyclocreatine inhibition caused a significant reduction of actin accumulation in the phagocytic cup area, and reduced complement receptor–mediated, but not Fc-γR–mediated, ingestion capacity of macrophages. Finally, we found that inhibition of CK-B affected phagocytosis already at the stage of particle adhesion, most likely via effects on actin polymerization behavior. We propose that CK-B activity in macrophages contributes to complement-induced F-actin assembly events in early phagocytosis by providing local ATP supply.

## Author Summary

To do work, cells need energy in the form of ATP. High and sudden energy demand is seen during cell-shape change, a process in which ATP fuels the cytoskeletal machinery that drives cell-morphology alteration. How a cell organizes high-energy surges without disrupting global ATP homeostasis remains an important research question. One view proposes that ATP is heterogeneously distributed, but the cytoskeletal proteins actin and myosin receive regional and preferential access to ATP. Yet this model raises another question: how is ATP funneled to these proteins from distant sources? To address some of these questions, we studied the highly localized molecular events controlling actin dynamics around phagocytic activity of macrophages. We demonstrate that actin and creatine kinase-B (CK-B), a long-known enzyme involved in ATP supply, are simultaneously recruited into the sites of action during the early phases of particle ingestion. Local availability of CK-activity and local generation of ATP promotes on-site actin remodeling and particle capture efficiency, and thus supports successful initiation of the first phases of phagocytosis. Interestingly, this coupling between local CK-activity and actin regulation is only relevant for complement-mediated phagocytosis (used by immune cells to target specific particles for ingestion). We predict that our findings may also shed light on how shape dynamics is energized in other cell types.

## Abstract 3

A tight connection exists between local recruitment of creatine kinase-B (CK-B) and actin remodeling activity in phagocytic cups of macrophages. Complement-mediated phagocytosis is stimulated in the presence of enzymatic-active CK-B, indicating that local ATP supply stimulates actin-driven particle uptake events.

## Introduction

Dynamic reorganization and stabilization of the actin cytoskeleton and membrane-shape alterations of cells are intimately and reciprocally coupled events that are essential for a variety of distinct cell functions such as adhesion, motility, cytokinesis, and endocytosis [1]. One of the processes that is critically dependent on proper regulation of actin polymerization is phagocytosis, essential for food intake in lower eukaryotes or the elimination of invading microbial pathogens and scavenging of dead cells in higher multicellular eukaryotes [2]. Engulfment of a phagocytic target is a spatially confined process, which is initiated at the cell membrane by recognition of the molecular structure at the surface of the phagocytic targets by dedicated receptors, such as Fc-gamma receptors (Fc-γRs), mannose receptor, or the complement receptor 3 (CR3, Mac-1) [3–5]. After binding of the target, receptors cluster, become activated, and trigger actin-dependent cytoskeletal changes via the activation of small Rho GTPases and concomitant induction of specific protein kinase signaling cascades [2,6]. In Fc-γR–mediated phagocytosis, signals are mediated through Rac and Cdc42, whereas CR3-regulated phagocytosis of complement-opsonized targets requires only RhoA activation [7]. These pathways ultimately converge and lead to the induction of Arp2/3-mediated actin polymerization, which is considered the main driving force for the formation of circular pseudopod protrusions (i.e., a “phagocytic cup”) around the target [8]. Once the wrapping in cellular membrane protrusions is complete, a contractile force is generated to engulf the particle or dead cell completely and guide the contents of the vesicle into the endocytotic pathway for degradation [9].

All events during early phagocytosis, including ruffle formation, membrane delivery, closure of the phagocytic cup, and short-range movement of newly formed vesicles through the cellular cortex, depend on actin polymerization and myosin motor proteins. In turn, for proper regulation of polymerization of G-actin into F-actin, which involves filament nucleation and extension, a spatially confined supply of ATP for the loading of actin subunits is required [10–12]. Theoretical models predict that ATP primarily promotes an “adjusted fit” of incoming monomers to the end of the actin filaments, and multiple studies agree that the ATP/ADP loading state of actin and related Arp2/3 proteins determine filament assembly or branching behavior [1,13,14]. Moreover, during filament severing or turnover, energy is used when ATP is hydrolyzed when still bound to F-actin, and P_i_ is released. Next, ADP-actin dissociates, and free G-actin monomers can be subsequently reloaded with “new” ATP. ATP- or ADP-loaded actin monomers are both competent for polymerization, but the nature of the bound nucleotide differentially modulates the kinetics of the association and dissociation at the pointed or barbed ends of filaments. Also the “storage” of G-actin monomers into thymosin- or profilin-sequestered pools is dependent on nucleotide loading state and hence, the energy state of the cell [12,15]. Active recruitment of G-actin and F-actin dynamics thus consumes ATP in several steps, and the active cell-shape remodeling needed for particle ingestion renders phagocytosis a process with a very high local requirement for high-energy phosphoryl (∼P) groups.

In fact, the energy dependence of phagocytosis is made even more prominent, because ATP is also necessary to sustain the activity of several nonmuscle myosin ATPases, which help in actin and membrane recruitment, and provide motor activity around the phagocytic cup [16,17]. For example, myosin-II activity is implicated in phagocytic cup formation and squeezing [18,19], whereas myosins X and VII may have roles in pseudopod extension and phagosome internalization [17,19–21]. By forming an ATP drain for these many actomyosin-based micromechanical events, phagocytosis may thus pose a formidable challenge to cellular energy homeostasis. Indeed, metabolic studies report increased energy turnover during phagocytosis [22,23].

Creatine kinase (CK)-mediated phosphotransfer plays an important role in local delivery and cellular compartmentation of ATP and transport from glycolytic or mitochondrial production sites [24,25]. The CK reaction buffers ATP and ADP levels by the reversible transfer of high-energy phosphoryl onto creatine (Cr) to form phosphocreatine (PCr): MgATP^2-^ + Cr ↔ MgADP^−^ + PCr^2-^ + H^+^ [26]. In muscle, localized delivery of ATP by the muscle-type creatine kinase (CK-M) isoform is clearly of importance for sustenance of acto-myosin ATPase activity involved in myofibrillar sliding activity during repeated high-speed contraction [27]. In brain, we [28] have obtained evidence that lack of brain-type CK (CK-B) activity affects synaptic coupling efficiency, a process for which active actin remodeling is essential [29]. By analogy, we hypothesized that the functional coupling between actin-based cytoskeletal dynamics and CK-mediated ATP compartmentalization and supply could be more general, and might be of importance for shape changes and dynamics of nonmuscle or neuronal cells as well. Here, we confirm this view and report on the role of the CK-PCr system in the dynamics of phagocytosis. Interestingly, Loike et al. [23] have found that brain-type CK is expressed in macrophages and that PCr levels decrease during phagocytosis. Our data suggest that the metabolic ATP-supply activity of CK-B is of local importance and facilitates specific phagocytosis steps via effects on actin-based events early in the binding-ingestion process.

## Results

### Endogenous CK-B Translocates to Phagocytic Cups in Microglia and Macrophages

**Figure 1:**
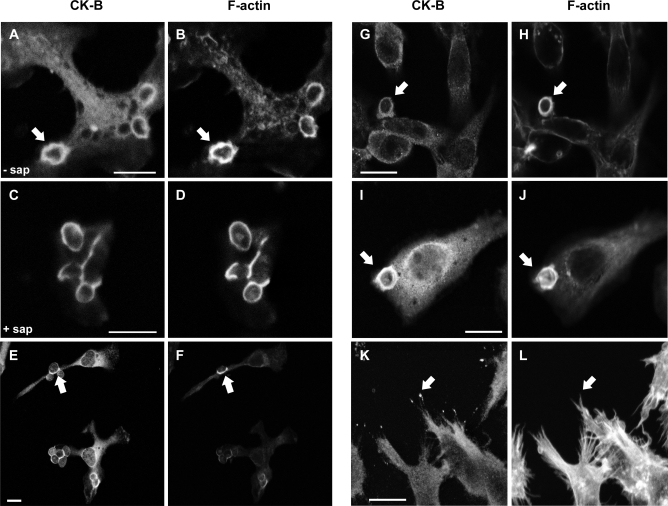
Cytosolic CK-B Accumulates in the Phagocytic Cup Area of Macrophages Uptake of zymosan in primary microglia (A–D), primary peritoneal macrophages (E and F), and RAW 264.7 macrophages (G–J). Fixation followed by (immuno)staining with CK-B antibodies (A, C, E, G, I, and K) or phalloidin (B, D, F, H, J, and L) reveals the co-accumulation of CK-B and F-actin at the phagocytic cup (arrows). (C and D) Saponin (sap) extraction of phagocytosing microglia prior to fixation and CK-B or actin staining (I–L) RAW 264.7 macrophages overexpressing mouse CK-B. Note that CK-B shows additional pronounced spot-like accumulation at the distal tips of filopodia (arrows, [K and L]). Bar represents 10 μm.

Phagocytic cup formation is characterized by a localized expansion of the plasmalemmal membrane, coupled to highly active remodeling and myosin-based contraction of the actin cytoskeleton. We studied the possible fate and role of endogenous CK-B in this process, in primary microglia and peritoneal macrophages after induction of phagocytosis with nonopsonized zymosan. Macrophages and microglia [2,30] are cells of the immune system that are very active in ruffle extension and uptake of extracellular particles. Although it has been reported that primary macrophages express CK-B [31], no data are available on the enzyme's behavior under conditions of active phagocytosis. Figure 1A and 1E shows that a fraction of CK-B always remained diffusely distributed throughout the cytosol, as in nonphagocytosing cells, but that a substantial portion of CK-B accumulated around the engulfed zymosan particles at nascent phagosomes. This accumulation did not occur exactly simultaneously in all cells because phagocytosis was not initiated fully synchronously throughout the culture, but at later time points, the concentrated staining dissipated (unpublished data), indicating that CK-B associated only transiently with phagosome structures.

Phalloidin staining demonstrated an almost complete overlap with CK-B encircling the zymosan particles in the phagosome (Figure 1B and 1F). To assess whether CK-B is actually locally bound within the cup area, microglia were permeabilized with saponin before fixation to remove most of the unbound cytosolic protein. Strikingly, a fraction of endogenous CK-B remained associated with the actin-rich area (Figure 1C and 1D). These data indicate that part of the CK-B molecules in the endogenous pool partition into sites of active F-actin remodeling.

To further verify the general validity of this picture, we analyzed the behavior of endogenous or exogenously transfected CK-B in the murine macrophage cell line, RAW 264.7. As anticipated, we also observed in this cell a uniform cytosolic distribution of endogenous CK-B and coaccumulation with F-actin at nascent phagosomes (Figure 1G and 1H). To compensate for the rather weak endogenous CK-B staining in RAW 264.7 cells, we also produced pools of cells with a higher CK-B steady-state level by transduction with retroviral vectors to enhance immunofluorescent detection. Again, prominent accumulation of CK-B together with F-actin appeared in the phagosome (Figure 1H and 1J). Notably, in RAW 264.7 cells with an overall high global CK-B level, we noticed CK-B accumulation at the distal tips of filopodia (Figure 1K and 1L). This phenomenon has been observed for a number of other proteins involved in cytoskeletal rearrangement, dynamic adhesion, and phagocytosis, including myosin-X, myosin-VII, and vasodilator-stimulated phosphoprotein (VASP) [20,21,32].

### CK-B Is Transiently Recruited to the Phagocytic Cup

**Figure 2:**
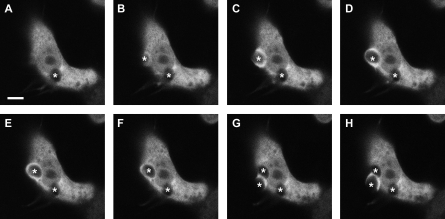
Transient Recruitment of EYFP-Tagged CK-B Time-lapse microscopy of zymosan uptake in RAW 264.7 cells transiently transfected with EYFP-tagged CK-B. Eight subsequent images captured at 6-s intervals over a 48-s recording period are shown. Asterisks indicate zymosan particles binding at the cellular membrane, during the process of engulfment, and after internalization. Bar indicates 5 μm.

As CK-B's role might involve the delicate interplay between compartmentalized energy supply and local molecular dynamics in the cell cortex area, we monitored the profile and timing of CK-B recruitment at the phagosome in more detail. To obtain dynamic information, we transiently expressed enhanced yellow fluorescent protein (EYFP)-tagged CK-B (via N-terminal fusion) in RAW 264.7 cells, and applied live cell microscopy imaging after induction of phagocytosis. Earlier work showed that N-terminal tagging of CK-B does not affect its enzymatic or structural properties [33]; (unpublished data). In the first (Figure 2A) of eight sequential frames of a movie (see Video S1) of typical CK-B behavior in an active macrophage, one particle is already being internalized (indicated by an asterisk), but at that point in time, EYFP-CK-B appears nonpartitioned and is still diffusely distributed throughout the cytoplasm. In subsequent frames (Figure 2B–2F), a clear CK-B accumulation in the phagocytic cup is observed, dissipation of which occurs when the particle is fully internalized (Figure 2G). A second phagocytic event with recruitment is initiated in the same cell at a later time point (Figure 2G and 2H). In control cells expressing nonfused EYFP, no significant accumulation at the site of zymosan ingestion was ever seen. A relatively straightforward interpretation of these observations would be that the spatially confined recruitment of CK-B serves phagocytic cup formation and/or closure, presumably via local delivery of ATP.

### Inhibition of CK-B Diminishes Actin Accumulation in the Phagocytic Cup

**Figure 3:**
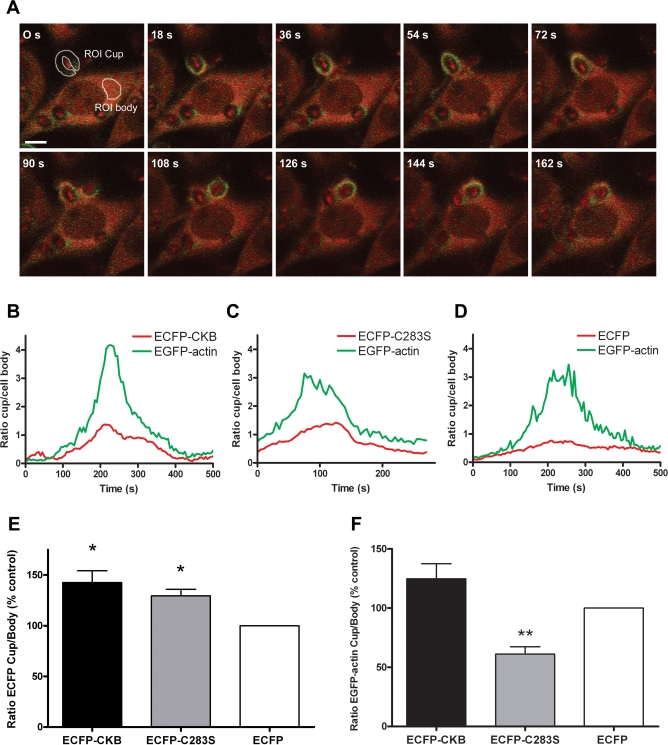
CK-B_C283S_ Decreases Actin Accumulation in Phagocytic Cups (A) Time-lapse images of a cell coexpressing EGFP-actin and ECFP-CK-B in the process of internalizing a zymosan particle, demonstrating the simultaneous accumulating signals from ECFP-CK-B (red) and EGFP-actin (green). Representative regions of interest (ROIs) are being shown for the phagocytic cup (ROI cup) and the cytosol (ROI body). Bar indicates 5 μm. (B–D) Signal intensities in the phagocytic cup and cell body were analyzed in cells coexpressing EGFP-actin and ECFP-CK-B, ECFP-CK-B_(C283S)_, or ECFP. The average pixel intensities in the ROIs were determined and the cup/body ratios for EGFP-actin (green) and ECFP-CK-B, ECFP-CK-B_(C283S)_, or ECFP (red) plotted against time. (E) Average maximal cup/body ratios for 10–16 events in cells expressing ECFP-CK-B, ECFP-CK-B_(C283S)_, and ECFP. (F) Average maximal EGFP-actin cup/body ratios in the same cells as in (E). Bars depict mean value with error bars representing the standard error of the mean (SEM). Number of events analyzed: *n* = 16 for ECFP-CK-B; *n* = 10 for ECFP-CK-B_(C283S)_ and *n* = 13 for ECFP. *p < 0.01; **p < 0.005.

A hallmark of phagosome formation is the rapid polymerization of F-actin, which drives the membrane extension around the target. Also actomyosin motor sliding is intimately coupled to this process [17,19–21]. To study whether these processes are indeed among the ones served by local CK-B activity, we compared zymosan-driven phagocytosis in RAW 264.7 cells that were stably coexpressing enhanced green fluorescent protein (EGFP)-tagged β-actin and either enhanced cyan fluorescent protein (ECFP) alone, ECFP-tagged CK-B, or a mutant CK-B_(C283S)_. This latter CK variant acts as a dominant-negative enzyme, occurring as a normal dimer with only 4% residual kinase activity [34]. Parallel spectral monitoring of fluorescence intensities enabled us to follow simultaneously the dynamic behavior of actin and CK-B variants, or the ECFP control, after induction of zymosan-driven phagocytosis (Figure 3A). Plotting of local signal intensities in relation to the global intensities in the cell body, which remained constant and were comparable for all cells examined (unpublished data), showed that recruitment of ECFP-tagged CK-B and CK-B_(C283S)_ occurred in nearly identical spatiotemporal overlap with EGFP-actin recruitment in all cases examined (Figure 3B and 3C; 10–16 individual events analyzed).

As anticipated, the average maximal cup/body signal ratio for ECFP-CK-B and ECFP- CK-B_(C283S)_ (142% ± 47% and 129% ± 21% , respectively) was significantly higher (*p* < 0.005) than that for ECFP alone (100%; Figure 3E). Thus, mobilization of CK-B protein to the phagosome area does not appear to be dependent on CK enzymatic activity. All cells examined displayed a clear accumulation of actin in the cup during a typical phagocytic event as defined by an increasing cup-to-body ratio of the EGFP signal (EGFP_Cup_/EGFP_Body_ > 1) (Figure 3B–3D). Strikingly, this accumulation differed significantly between cells expressing ECFP-CK-B or ECFP-CK-B_C283S_ (Figure 3F), and for the ECFP- CK-B_(C283S)_ cell line, was markedly decreased (61% ± 18%) compared to ECFP cells (*p* < 0.005). Cells in an independently generated pool harboring the ECFP-tagged CK-B_(C283S)_ exhibited a similar decrement, demonstrating that the observed decrease was not cell line or pool specific (unpublished data). In ECFP-CK-B cells, the green actin signal reached a slightly higher maximal cup/body ratio of 117% ± 51% than in ECFP-control cells (100%). This difference was not significant, however, and we therefore consider it the result of experimental variation.

To establish whether effects of absence/presence of active CK-B affected the temporal profile of actin recruitment, we also compared the timing of actin mobilization between different movies of different cell transfectants. No significant differences were found. These results demonstrate that local presence of metabolically active CK-B alters the magnitude, but not the timing, of actin mobilization at the phagosome.

### CK-B Accumulates Independent of Type of Opsonization

**Figure 4:**
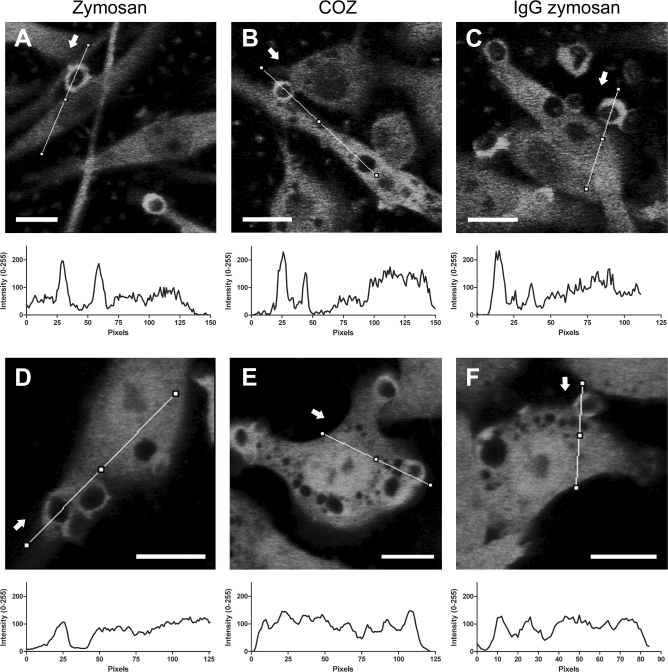
Dynamic Redistribution of EYFP-Tagged CK-B during Phagocytosis. Time-lapse microscopy of zymosan (A and D), COZ (B and E), or IgG-opsonized zymosan (C and F) uptake in RAW 264.7 cells stably transfected with EYFP-CK-B (A–C) or EYFP (D–F). Photos represent a single frame at the peak of accumulation from a time-lapse recording; arrows mark the start of the corresponding line plot visualizing accumulation of signal in the cup. Bars indicate 10 μm.

The molecular structure at the surface of the phagocytic target determines which receptor types become ligand bound and activated. Subsequently, receptor-specific downstream signaling events, such as alternative use of small Rho GTPases and kinases, orchestrate the outcome of the phagocytic process [7,19,35]. To test whether CK-B recruitment is a default response or determined by the surface properties of the target, we repeated our time-lapse experiments with cells that were challenged with native zymosan (Figure 4A), or zymosan opsonized with either complement (COZ) (Figure 4B) or immunoglobulin G (IgG) (Figure 4C). To avoid that effects of other properties of the target, such as rigidity and geometry, would influence the outcome of our study [36,37], we deliberately chose to change only the type of coat, not the particle type (i.e., zymosan) in these experiments. Line-plots of pixel intensities across the phagocytic cup and other areas of the cell body revealed that EYFP-CK-B recruitment occurred independently of the type of opsonization. Montages of control cells with untagged EYFP did not reveal any specific mobilization into or around phagocytic cups (Figure 4D–4F). These data are consistent with the idea that spatially confined presence of CK-B in the cup area is interlinked with general steps in phagocytic cup formation and/or closure.

### Cyclocreatine Inhibits Phagocytosis of Zymosan and COZ

**Figure 5:**
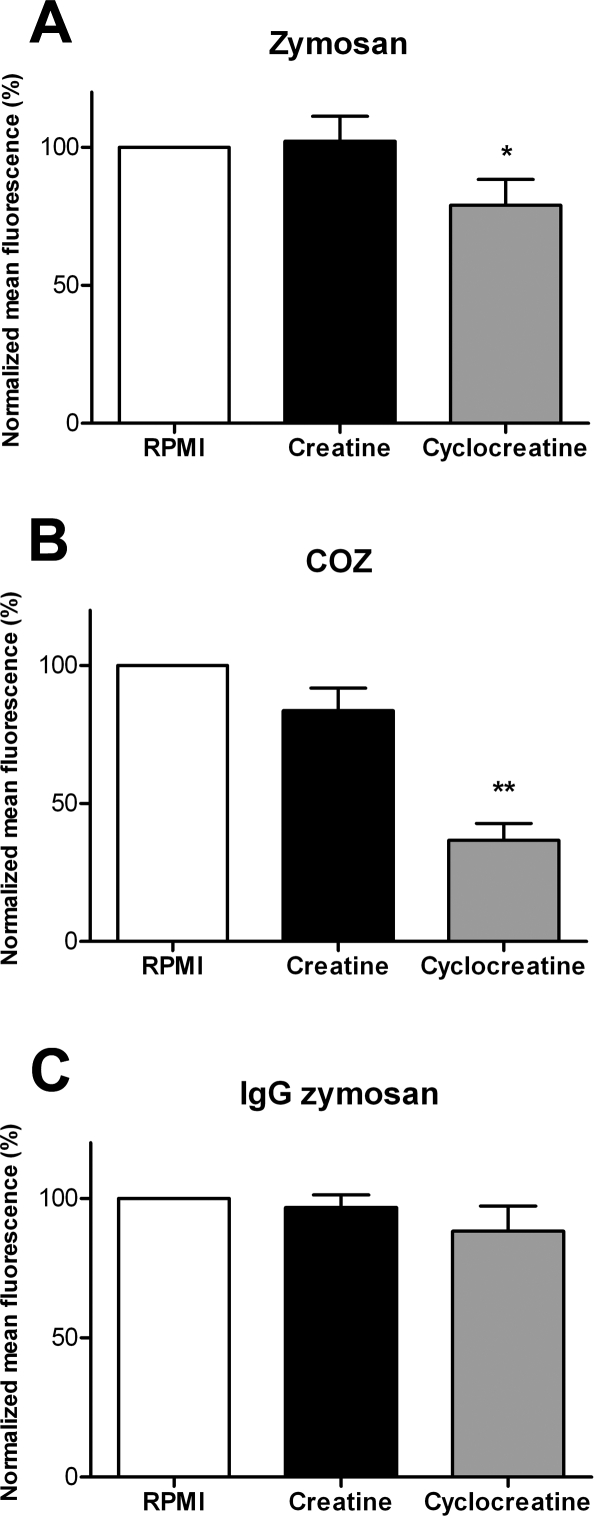
Cyclocreatine Inhibits Phagocytosis Fluorescent particle uptake capacity quantified by FACS in RAW 264.7 cells preincubated with 5 mM creatine, 5 mM cyclocreatine, or normal growth medium. (A) Zymosan. (B) COZ. (C) IgG-opsonized zymosan. Bars represent averages of three to four experiments performed in duplicate (±standard deviation [SD]). *p < 0.03; **p < 0.005.

On the basis of this premise, we wondered whether the CK-driven ATP–PCr exchange reaction could be directly or indirectly coupled to the process of particle ingestion. Initially, we chose a pharmacological approach to modulate activity of the entire cellular pool of CK, applying Cr as a stimulating substrate or cyclocreatine (cCr) as a reversible inhibitor of the CK reaction. RAW 264.7 cells were preincubated with 5 mM cCr or Cr prior to the phagocytosis assay, and cells were then challenged with differentially opsonized and fluorescently labeled phagocytic targets. After 30 min, phagocytic activity was quantified by determining the mean fluorescence intensity of ingested particles in the different cells by fluorescence-activated cell sorter (FACS) analysis. In Figure 5, data are shown that are normalized to values for nontreated cells. Phagocytosis of nonopsonized zymosan was slightly affected by cCr treatment, yielding an efficiency value of 79% ± 9% (*p* < 0.05), whereas Cr supply had no significant effect (102% ± 9%) (Figure 5A). Interestingly, cCr inhibition decreased phagocytosis of COZ to a much lower level, 37% ± 6% (*p* < 0.005) of that of nontreated cells, whereas Cr addition had no significant effect (value 84% ± 8%) (Figure 5B). In contrast, Cr and cCr addition had no significant effect on phagocytosis of IgG-opsonized zymosan, with 97% ± 5% and 88% ± 9% for Cr- and cCr-treated cells, respectively (Figure 5C). In order to verify that this difference was indeed due to differential effects on CR3 and Fc-γR receptor-mediated activities and cannot be attributed to interference with other pathways, we performed receptor-blocking experiments. Capture uptake of the two different types of opsonized zymosan appeared indeed specific for the anticipated receptors (Figure S1). To study this point further, we also tested phagocytic activity on complement- and IgG-opsonized polystyrene beads, which lack obvious surface ligands such as mannose or β-glucan groups, and therefore form “clean” targets. Interestingly, uptake of complement-opsonized beads was again inhibited by cCr (56 ± 4% of control), whereas IgG-mediated phagocytosis remained unaffected (95 ± 2% of control; Figure S2).

Thus, although CK mobilization is seemingly a default event in all types of phagocytosis (Figure 4), it may only selectively contribute to the efficiency of phagocytic ingestion of nonopsonized or complement-opsonized particles (Figures 5 and S2). A similar situation was recently reported for the cytoskeletal actin-binding protein talin, whose functional role in phagocytic uptake appeared selectively coupled to CR3, but which accumulates in phagosomes formed around IgG- and C3-opsonized particles [38].

### RAW 264.7 Macrophages Expressing CK-B_(C283S)_ Exhibit Impaired Phagocytosis

**Figure 6:**
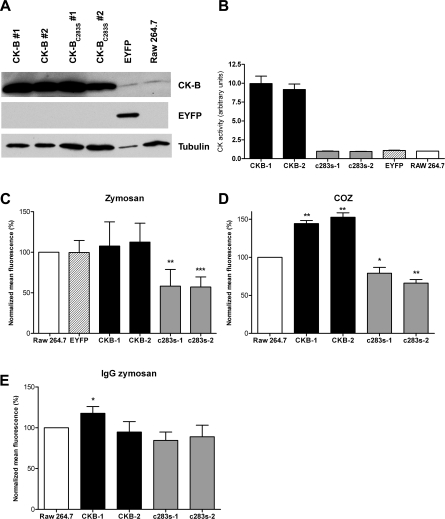
CK-B and CK-B_(C283S)_ Influence Phagocytosis (A) Western blot analysis of RAW 264.7 cells stably expressing CK-B or CK-B_(C283S)_. Two individual retrovirally transduced populations were maintained (CK-B#1 and CK-B#2, and CK-B_(C283S)_#1 and CK-B_(C283S)_#2). RAW 264.7 cells expressing EYFP and noninfected cells were used as control. (B) CK enzymatic activity of depicted cell lysates. Fluorescent particle uptake capacity quantified by FACS in cell lines incubated with zymosan (C), COZ (D), or IgG zymosan (E). Bars represent the average of three to four experiments (±SD). *p < 0.05; **p < 0.005; ***p < 0.0005.

To address CK-B's specific role in phagocytic activity in another manner, we also compared effects of expression of the CK-B_(C283S)_ mutant and that of CK-B. To obtain comparable levels of expression of these proteins across all individual cells in and between cell populations, transduction with retroviral vectors encoding CK-B, the CK-B_(C283S)_ mutant, or EYFP was used (resulting cell pools are hereafter referred to as RAW-CK-B, RAW-CK-B_(C283S)_, and RAW-EYFP cells, respectively). Two independent cell pools were established for each construct to rule out potential integration-site–dependent effects and/or effects of overgrowth of specific cell clones. Western blotting was performed to assess expression levels in our stable cell lines (Figure 6A). The levels of the exogenously expressed wild-type (wt) or mutant CK-B protein in the RAW-CK-B or RAW-CK-B_(C283S)_ cell pools amounted to roughly ten times more than the endogenous CK-B level in these cells. The total enzymatic phosphoryl transfer activity had increased accordingly, and approximated a 10-fold higher steady-state level in both RAW-CK-B cell populations relative to the reference RAW 264.7 cell pool (Figure 6B). As anticipated, the total CK activity of the control line expressing EYFP matched that of the endogenous activity in nontransduced control cells. Also, both pools of RAW-CK-B_(C283S)_ mutant cells exhibited levels of CK activity that were identical to endogenous levels. Clearly, the residual activity for the mutant CK-B_(C283S)_ is very low, and therefore no appreciable increase in CK activity was noticeable despite the almost 10-fold increase in protein level.

Assessment of phagocytic capacity with fluorescently labeled zymosan and COZ confirmed that neither the retroviral infection nor the subsequent antibiotic selection procedure had affected phagocytic capacity, since RAW-EYFP cells and noninfected cells behaved identically (Figure 6C). Expression of CK-B_(C283S)_, however, resulted in a considerable drop in phagocytosis efficiency. Uptake of nonopsonized zymosan was at 58 ± 21% and 57 ± 12% (*p* < 0.005) for both RAW-CK-B_(C283S)_#1 and RAW-CK-B_(C283S)_#2, respectively, relative to nontransduced or EYFP-expressing cells. We observed that overexpression of CK-B had no stimulating effect and did not significantly alter phagocytosis of zymosan (108 ± 30% and 113 ± 23% for the two independent cell lines). In contrast, in the case of complement-mediated (COZ) phagocytosis, both RAW-CK-B cell pools did perform significantly better than controls (144 ± 4% and 153 ± 6%, *p* < 0.005) (Figure 6D). Conversely, the cell lines expressing CK-B_C283S_ were also significantly impaired in the uptake of COZ (79 ± 8% and 66 ± 5%, *p* < 0.05). Thus, expression of CK-B_(C283S)_ impaired phagocytosis of both zymosan and COZ, whereas overexpression of wt CK-B stimulated only the phagocytosis of COZ. Importantly, expression of CK-B_(C283S)_ did also not influence IgG mediated phagocytosis (Figure 6E; efficiency of 85 ± 10% and 89 ± 14% for both lines), in line with our results with pharmacological inhibition. Unfortunately, our findings of effects of overexpression of wt CK-B on IgG-mediated phagocytosis were inconclusive. One cell line displayed a moderate increase in phagocytotic efficiency (118 ± 8%; *p* < 0.05), whereas the other did not differ significantly from the control (95 ± 13%). Although identical cell pools were used for the experiments shown in Figure 6C–6E, the experiments presented in Figure 6E were performed with cells at a higher passage number. We therefore may have to attribute the borderline stimulation to an effect unrelated to CK-B.

Because total cellular CK activity was identical between the parental RAW264.7 line and RAW-CK-B_(C283S)_, and in addition, CK-B_(C283S)_ is recruited in a similar fashion as wt CK-B (Figure 3), our findings suggest that the mutant protein competes with endogenous CK-B and thereby lowers the concentration of locally active CK-B molecules at crucial sites in the cell cortical area.

### RAW 264.7 Macrophages Expressing CK-B_(C283S)_ Exhibit Impaired Adhesion and Internalization of COZ

**Figure 7:**
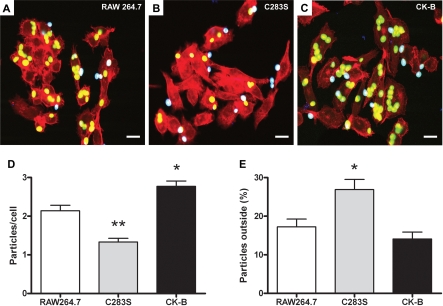
CK-B Is Involved in the Initial Steps of Phagocytosis Adhesion and uptake of FITC-labeled COZ particles in RAW 264.7 cells (A) and RAW 264.7 cells expressing CK-B_(C283S)_ (B) or CK-B (C). Internalized particles appear green (or yellow, when colocalizing with F-actin), and external particles appear cyan. Bars indicate 10 μm. (D) Averages of total numbers of particles associated per cell (inside + outside) (±SEM). *p < 0.005; **p < 0.001. (E) Percentage particles attached to the cell, but not yet engulfed (±SEM). *p < 0.05.

Phagocytosis occurs through a series of consecutive steps that ultimately lead to the engulfment of a particle. Probing for adhesion of coat molecules, and the actual binding of the phagocytic target to specific receptors on the protruding cell surface, constitutes one of the first steps in this process. In order to specify which specific phase of the phagocytic process is linked to CK-B, we subjected the wt CK-B or CK-B_(C283S)_ cell pools to a particle adhesion assay, using COZ particles as the phagocytic targets with most discriminative effects (Figure 7). Quantification of the total number of particles per cell (inside + outside) in images of the cell lines with adherent and already internalized particles (Figure 7A–7C; *n* = 3 experiments) demonstrated that an average of 1.3 ± 0.7 of COZ particles associated with RAW-CK-B_(C283S)_ cells, significant less than with control cells, which have 2.1 ± 0.7 particles/cell (*p* < 0.001). Interestingly, RAW-CK-B cells bound significant higher numbers of particles than control cells (2.8 ± 0.7 particles/cell; *p* < 0.005). Calculation of the percentage of external COZ particles revealed that control cells have 17 ± 10% of particles attached that are not yet (fully) internalized. Overexpression of CK-B did not affect this percentage (14 ± 9% external). With RAW-CK-B_(C283S)_ cells, a significantly higher percentage of particles remained external (27 ± 18% ; *p* < 0.02). Inhibition of CK-B thus apparently affects both the initial sampling of COZ particles from the added pool as well as well as the process of their internalization.

### CK-B Facilitates Actin Polymerization

**Figure 8:**
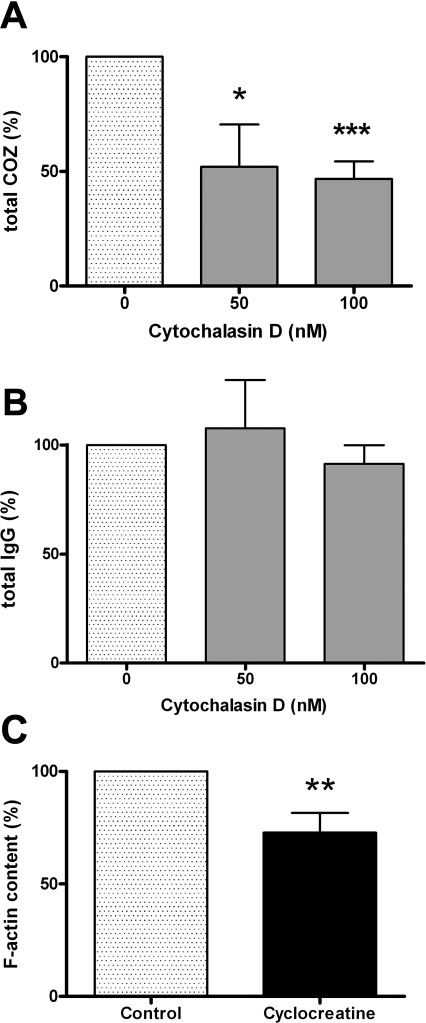
Coupling between CK-B Activity, Actin Polymerization State, and Phagocytosis Adhesion assays were performed with RAW 264.7 cells, pretreated with cytochalasin D (50 or 100 nM) and COZ (A) or IgG-opsonized zymosan (B). The total number of particles (adherent and internalized) was determined and normalized to nontreated cells (averages of 3–4 experiments ± SD). (C) Raw 264.7 cells were treated with 5 mM cCr prior to F-actin quantification using fluorescently labeled phalloidin (averages of four experiments ± SD). *p < 0.05; **p < 0.01; ***p < 0.001.

Recently, it has been demonstrated that cells actively probe the extracelullar matrix for adhesion sites by clustering integrins in “sticky fingers” at the leading edge of cells. Actin polymerization has an active role in this process [39]. Since extension–retraction of filopodial tentacles that determine the efficiency of particle uptake in phagocytosis is also based on F-actin in combination with myosin-V, -VII, and -X activities [40], we decided to study whether the role of F-actin in adhesion of COZ and IgG-opsonized zymosan (Figures 5B and 5C, and 6D and 6E) in RAW 264.7 cells could be different and correlated with the differential effects of CK-B. Therefore, adhesion experiments with COZ and IgG-opsonized zymosan particles in the presence of low concentrations of the actin polymerization inhibitor cytochalasin D were performed (Figure 8A and 8B). Interestingly, treatment with 50 nM or 100 nM cytochalasin D decreased adhesion of COZ dramatically (52 ± 18%; *p* < 0.05; and 47 ± 8%; *p* < 0.001, respectively; *n* = 4; Figure 8A), whereas adhesion of IgG-opsonized zymosan was not significantly affected (108 ± 22% and 91 ± 9% for 50 nM and 100 nM cytochalasin D, respectively; *n* = 3; Figure 8B). We consider this evidence for a different role of F-actin in complement- and IgG-mediated adhesion.

To further address whether this discriminative coupling could indeed be linked to CK-B's role in providing adequate ATP supply for F-actin formation [41,42], RAW 264.7 cells were treated with cCr, and the F-actin content was determined. Fluorescent phalloidin staining in combination with FACS analysis revealed that in cCr-treated cells, the global F-actin content significantly decreased to 73 ± 9% (*p* < 0.01, *n* = 4) of nontreated control cells (Figure 8C). Thus, inhibition of CK-B–mediated activity indeed affects the formation of F-actin in RAW 264.7 cells. This is in agreement with our finding that actin recruitment to phagocytic cups is also diminished when CK-B is inhibited (Figure 3F). The question whether there is also a reciprocal relationship, whether the local F-actin state contributes to CK-B recruitment to the phagocytic cup, appeared more difficult to answer. Until now, we were unable to detect a direct binding between actin and CK-B in pull-down experiments (unpublished data). Furthermore, fluorescence recovery after photobleaching (FRAP) experiments revealed that the motility of YFP-actin and CFP-CK-B in cup areas differed during the phagocytic process (Figure S3), arguing against single association between actin and CK-B. Involvement of transient “kiss-and-run” type interactions cannot be excluded, however.

Combined, our data suggest that the activities of CK-B that we have described are likely to occur via ATP-supply effects on local F-actin polymerization capacity, which in turn affects CR3-mediated adhesion and internalization.

## Discussion

Phagocytosis requires a rapid and spatially confined reorganization of the actin cytoskeleton. The underlying molecular processes, such as actin polymerization and actomyosin force generation, generate a sudden and localized demand for cellular ATP [22,23]. To reciprocate this challenge, sites of ATP production should be coupled tightly to sites of ATP consumption. From studies in cell and animal models, we know that CK isozymes are particularly well equipped for this role, as they provide the cell with a fast ATP regeneration and delivery system that can adequately provide high-energy phosphoryl groups to cellular locales with high energy turnover. Here, we established a tight functional and spatial link between the CK system and the actin-based cytoskeletal machinery in macrophages during phagocytosis. A similar relationship was found for the muscle isoform of CK, CK-M, which associates with the M- and I-bands of skeletal muscle and fuels local ATP-consuming processes, including actomyosin contraction and calcium pumping [26,43]. Importantly, CK-M's role in these physiological processes is supportive, not absolutely vital [27,43], just as we report here for CK-B's role in phagocytosis. For CK-M, the molecular nature of events that support its role has been unraveled to some extent. For example, we know that interaction of CK-M with the sarcomeric M-band is mediated via conserved lysine residues. In addition, particular amino acid segments in CK-M enable the protein to bind indirectly to the I-band via the glycolytic enzymes phosphofructokinase (PFK) and aldolase, which have actin-binding properties [44]. Interestingly, glycolytic enzymes are also known to be recruited to phagosomes [45,46], so there may be parallel mechanisms. Unfortunately, to explain CK-B dynamics, it is not possible to use simple analogy since the lysine residues involved in CK-M M-band interaction are not conserved in CK-B [47], and direct sequence comparison is not yielding clear clues for other binding modes—for example to glycolytic enzymes. Several mechanisms could therefore be involved in the recruitment of CK-B. One model would be the transient availability of CK-B binding sites at the nascent phagosome by modification of local proteins or presence of CK-B interacting proteins. Based on its colocalization with CK-B, we tested whether actin could be a candidate for such scaffolding, but pull-down assays and FRAP experiments did not reveal a tight interaction. Also yeast two-hybrid assays did not disclose any CK-B to actin binding opportunities (unpublished data). As another possibility, CK-B binding characteristics could also be transiently modified at the enzyme itself, possibly by (enzymatic) events located at the forming cup. Indeed, CK-B is prone to covalent modifications such as phosphorylation [48], oxidation [49], methylation [50], or ubiquination [51]. Simple presence of substrate may also determine binding ability, as recently shown for CK-M [52]. Further studies are necessary to discriminate between all these possibilities.

Because phagocytosis is a metabolically demanding process, CK-B recruitment to phagocytic cups could serve to promote or safeguard local events or—reciprocally—shield the rest of the cell from excessive local energy demand. To distinguish between these mechanistic models and elucidate CK-B global and local physiological role(s) in macrophages more precisely will be technically challenging because of the confined character of the events. This, therefore, also remains a topic for future study.

Of particular importance was our finding that displacement of endogenous CK-B by ECFP-CK-B_(C283S)_ during phagocytosis reduced the local accumulation of EGFP-actin in the phagocytic cup area. The observation that inhibition of CK-B activity impairs global F-actin polymerization in RAW 264.7 cells was equally revealing. Normally, actin polymerization requires the incorporation of ATP-actin at the barbed end of actin filaments. During filament elongation, ATP is hydrolyzed and ADP-actin is being released at the pointed end. Thus, constant reloading of actin with ATP is required for the continuation of the polymerization cycle [12,41,42]. We propose here, that CK-B could specifically enhance this process by regenerating ATP at sites of active actin remodeling.

In addition to the polymerization reaction, actin nucleation and branching play an important role. The Arp2/3 complex, together with the Wiskott-Aldrich syndrome (adapter) protein, WASP [8], and the motor proteins myosin-I [53,54] and myosin-II [19], helps to guide these processes during shaping of the cup of nascent phagosomes. Recently, the involvement of the formin mDia has also been implicated in phagocytosis of COZ. Inhibition of mDia results in decreased F-actin recruitment at the phagocytic cup and induces a concomitant decrease in efficiency of CR3-mediated, but not Fc-γR–mediated, phagocytosis [55]. Formins promote actin polymerization by increasing polymerization-associated ATP hydrolysis up to 15 times via profilin [56]. Finally, regulation of cell structure via RhoA activation or AMP-activated kinase involvement is also directly energy dependent [57,58]. Thus, different types of actin regulatory processes form local and temporal energy drains, which may need compensation by CK-B–mediated ATP regeneration.

Various associated myosin motor mechanisms involved in formation of the specialized structures at the phagosome may also be CK-B dependent, because motor activity of myosins is controlled by ATP/ADP ratio. In *Dictyostelium*, myosin-VII was found to be important for initial adhesion in phagocytosis [21]. In addition, another closely related myosin, myosin-X, was implicated in adhesion and phagocytosis [20,59]. However, at this point, we need more mechanistic information about how ATP fuelling separately serves actin and myosin activities in the phagocytic cup before we can analyze CK-B's presumed role(s) in detail further.

Intriguingly, Olazabal and coworkers [19] have reported that inhibition of myosin-II decreased actin recruitment in phagocytic cups during CR3-mediated phagocytosis, but not Fc-γR–mediated events. Our results regarding differences in ingestion capacity for COZ or IgG-coated zymosan and beads after genetic blocking or pharmacological inhibition of CK activity fit with a model in which ATP-driven actomyosin activities differentially contribute to discrete phagocytic processes. Only phagocytosis of COZ and zymosan was significantly affected under conditions in which CK activity was lowered, Fc-γR–mediated activity was not significantly affected by cyclocreatine or dominant-negative CK inhibition. Of note, phagocytosis of COZ is mainly, but not exclusively, mediated by complement molecules present on COZ. Also, sugar residues that are present on (non)opsonized zymosan facilitate recognition by lectin domains in CR3 [60–62] or alternative phagocytic receptors such as dectin-1 [63].

Based on our findings and these background notions regarding differences in pathways involved in cup formation in complement or IgG modes of phagocytosis [7,19,55,64], it is tempting to speculate that CK-B enhances phagocytosis by modulating specific processes up- or downstream of CR3. It may be important to note that differences in the actin levels in the phagocytic cup, as seen between active ECFP-CK-B and mutant ECFP-CK-B_(C283S)_–expressing cells, correlated better with the fraction of cells participating in phagocytosis than with the number of fully ingested particles per cell. The observation of the apparent delayed internalization of COZ particles in the RAW-CK-B_(C283S)_ line (Figure 7E) suggests that after early binding requirements have been fulfilled, CK-B also promotes transition to the next phases of phagocytosis. Therefore, we propose that the CK-B–mediated modulation of actin polymerization is particularly relevant during early CR3-mediated phagocytosis, for example by increasing the number of successful probing attempts for particle binding. Our data also suggest that endogenous levels of resident CK-B molecules are largely sufficient to saturate these requirements for zymosan and COZ phagocytosis.

CR3 is also known as CD11b/CD18 or αMβ2 integrin. Because actin polymerization is pivotal in early adhesion events mediated by integrins [39], a picture emerges in which actin behavior determines the efficiency by which CR3s can bind their target. Actin behavior may be less central for IgG-mediated binding events. Our finding that low concentrations of the actin polymerization inhibitor, cytochalasin D, reduced binding of complement opsonized particles more than IgG-opsonized particles is consistent with such a model.

In conclusion, we have demonstrated that CK-B enhances phagocytosis of zymosan and COZ, likely via a specific synergistic role in mechanistic events involved in actin polymerization behavior. Although our data indicate that the enzyme's metabolic role is dominant, we cannot completely rule out a structural role of CK-B in phagocytosis at this point. Taken together with the finding that CK-B_(C283S)_ was able to inhibit phagocytosis without decreasing the total CK activity, our data suggest that CK-B acts to steer the delicate local balance in ATP/ADP ratio, during formation of the phagocytic cup, around the time that pseudopod–filopodium extensions or CR3-mediated adhesions are formed. Because we do see CK-B in filopodia and phagocytic cups in our RAW cells (Figures 1G–1L and 4), but also observe CK-B accumulation in dynamic actin structures of other cell types during adhesion to substratum, spreading, and crawling, (e.g., in neurons and astrocytes; unpublished data), this raises the exciting possibility that CK-B facilitates rapid cytoskeletal dynamics in a broad range of specialized events that occur during tissue development and disease, including dendritic spine generation in brain [65], formation of immune-synapses, or protrusion dynamics for cancer cell invasion.

## Materials and Methods

### Cell culture.

Resident peritoneal macrophages were isolated from adult (10–20 wk old) mice of mixed genetic background (C57BL/6 × 129Ola). After sacrificing the mice by cervical dislocation, cells were harvested by rinsing the peritoneum twice with 5 ml of ice-cold HBSS. Collected macrophages were cultured for 24 h in RPMI supplemented with 10% fetal calf serum (FCS), glutamine (0.3 g/l), sodium pyruvate (0.11 g/l), and gentamycin (0.05 mg/ml). Primary microglia were collected from neocortices of newborn mice as described [66].

RAW 264.7 murine macrophages were maintained in RPMI 1640 (Gibco) containing 10% FCS, glutamine (0.3 g/l), sodium pyruvate (0.11 g/l), and gentamycin (0.05 mg/ml).

### Plasmid and retroviral expression vectors, transfection, and transduction.

Expression plasmids construction and transfection was done according to standard procedures. To generate catalytically inactive CK-B, cysteine-283 was replaced by a serine using the QuikChange Site-Directed Mutagenesis kit (Stratagene). Retroviral expression constructs were created by insertion of the ORFs of CK-B, CK-B_(C283S)_, and EYFP into retroviral vector pLZRS-IRES-zeo [67], giving rise to pLZRS-CK-B, pLZRS-CK-B_(C283S)_, and pLZRS-EYFP, respectively. Retroviral transduction was performed as described [67].

### Phagocytic targets and phagocytosis uptake assay.

As targets, unlabeled zymosan particles were used. Complement opsonization was performed as described [68], and cells were activated with 200 nM phorbol 12-myristate 13-acetate (PMA) 15 min prior to phagocytosis of COZ. IgG opsonization of zymosan was done using zymosan A BioParticles opsonizing reagent from Invitrogen.

For assay of phagocytic uptake efficiency, fluorescently labeled particles were added to 1 × 10^5^ RAW 264.7 cells at a ratio of ten particles per cell. The mean fluorescence of 1 × 10^4^ cells per sample was analyzed on a Becton-Dickinson FACScan flow cytometer.

### Indirect immunofluorescence and live cell imaging.

Indirect immunofluorescence was performed according to standard procedures. For live cell imaging, a Zeiss LSM510meta confocal laser scanning microscope was used. For dual imaging of RAW 264.7 cells stably expressing GFP-actin and ECFP constructs, spectral recordings were taken and separated using the linear unmixing option. Line plots were generated with ImageJ (National Institutes of Health) software.

### Immunoblotting and CK-activity assay.

Cell lysates were prepared using standard procedures. Immunoblotting was performed as described using the anti-CK-B antibody 21E10 [69]. CK activity was determined by an enzyme-coupled reaction.

### Adhesion assay.

Cells grown on glass cover slips were PMA stimulated (200 nM, 15 min), washed with serum-free RPMI, and incubated with FITC-labeled COZ particles (10 per cell) for 30 min at 37 °C. Cells were washed two times with PBS to remove nonbound particles, fixed and external zymosan was stained with anti-zymosan IgG (Molecular Probes). Cells were then permeabilized (3 min, 0.1% Triton X-100 in PBS) and incubated with Alexa660 conjugated goat anti-rabbit IgG and Alexa 568 conjugated phalloidin (Molecular Probes). The total number of particles and the number of external adherent particles per cell were calculated.

A detailed description of all procedures can be found as Protocol S1.

## Supporting Information

Figure S1 Determination of Receptors Used in Uptake of Phagocytic Particles The receptors on RAW 264.7 cells involved in binding of differentially opsonized zymosan particles were determined in the presence of a CR3 blocking monoclonal antibody (M1/70, anti-CD11b) (A and B) or human IgG (3 mg/ml) (C and D). Bar diagrams represent the percentage particles (internalized and bound) of COZ (A) and IgG-zymosan (B) normalized to the control.
(1.6 MB TIF)

Figure S2 Phagocytosis of Complement- and IgG-Coated Polystyrene Beads Uptake capacity for fluorescent polystyrene beads in RAW 264.7 cells preincubated with 5 mM creatine, 5 mM cyclocreatine, or normal growth medium was quantified by FACS.
(A) Complement-coated 3-μm polystyrene beads.
(B) IgG-coated 3-μm polystyrene beads. Bars represent averages of Fl-1–positive cells of two experiments performed in triplicate.
(1 MB TIF)

Figure S3 Determination of the Mobility of CK-B and Actin in the Phagocytic Cup (A) Average (*n* = 14) of normalized bleach and recovery curves of RAW264.7 cells stably expressing ECFP-CK-B and EYFP-actin. Fluorescence of CFP and YFP in the phagocytic cup was bleached by scanning a square region covering approximately half the cup area 20 times at high laser power.
(B) *T*_1/2_ of recovery (in seconds) of ECFP-CK-B and EYFP-actin in the phagocytic cup as determined from the individual recovery curves.
(373 KB TIF)

Protocol S1 Detailed Description of All Procedures (55 lB DOC)

Video S1 Movie of a EYFP-CK-B–Transfected Cell Phagocytozing Zymosan RAW 264.7 cells were transiently transfected with pEYFP-CK-B. Texas Red–labeled zymosan particles were added to the cells, and time-lapse microscopy was performed with an interval of 3 s between subsequent frames.
(1.2 MB MOV)

## Abbreviations

cCr: cyclocreatine
CK: creatine kinase
CK-B: creatine kinase brain-type
CK-M: creatine kinase muscle-type
COZ: complement-opsonized zymosan
Cr: creatine
CR3: complement receptor 3
ECFP: enhanced cyan fluorescent protein
EGFP: enhanced green fluorescent protein
EYFP: enhanced yellow fluorescent protein
FACS: fluorescence activated cell sorter
IgG: immunoglobulin G
PCr: phosphocreatine
wt: wild type

## References

[pbio-0060051-b001] Pollard TD, Borisy GG (2003). Cellular motility driven by assembly and disassembly of actin filaments. Cell.

[pbio-0060051-b002] Aderem A, Underhill DM (1999). Mechanisms of phagocytosis in macrophages. Annu Rev Immunol.

[pbio-0060051-b003] Linehan SA, Martinez-Pomares L, Gordon S (2000). Macrophage lectins in host defence. Microbes Infect.

[pbio-0060051-b004] Gessner JE, Heiken H, Tamm A, Schmidt RE (1998). The IgG Fc receptor family. Ann Hematol.

[pbio-0060051-b005] Ehlers MR (2000). CR3: a general purpose adhesion-recognition receptor essential for innate immunity. Microbes Infect.

[pbio-0060051-b006] Stuart LM, Ezekowitz RA (2005). Phagocytosis: elegant complexity. Immunity.

[pbio-0060051-b007] Caron E, Hall A (1998). Identification of two distinct mechanisms of phagocytosis controlled by different Rho GTPases. Science.

[pbio-0060051-b008] May RC, Caron E, Hall A, Machesky LM (2000). Involvement of the Arp2/3 complex in phagocytosis mediated by FcgammaR or CR3. Nat Cell Biol.

[pbio-0060051-b009] Vieira OV, Botelho RJ, Grinstein S (2002). Phagosome maturation: aging gracefully. Biochem J.

[pbio-0060051-b010] Dayel MJ, Holleran EA, Mullins RD (2001). Arp2/3 complex requires hydrolyzable ATP for nucleation of new actin filaments. Proc Natl Acad Sci U S A.

[pbio-0060051-b011] Jahraus A, Egeberg M, Hinner B, Habermann A, Sackman E (2001). ATP-dependent membrane assembly of F-actin facilitates membrane fusion. Mol Biol Cell.

[pbio-0060051-b012] Wolven AK, Belmont LD, Mahoney NM, Almo SC, Drubin DG (2000). In vivo importance of actin nucleotide exchange catalyzed by profilin. J Cell Biol.

[pbio-0060051-b013] Kovar DR (2006). Arp2/3 ATP hydrolysis: to branch or to debranch. Nat Cell Biol.

[pbio-0060051-b014] Becker EW (2006). The roles of ATP in the dynamics of the actin filaments of the cytoskeleton. Biol Chem.

[pbio-0060051-b015] Goldschmidt-Clermont PJ, Furman MI, Wachsstock D, Safer D, Nachmias VT (1992). The control of actin nucleotide exchange by thymosin beta 4 and profilin. A potential regulatory mechanism for actin polymerization in cells. Mol Biol Cell.

[pbio-0060051-b016] Allen LH, Aderem A (1995). A role for MARCKS, the alpha isozyme of protein kinase C and myosin I in zymosan phagocytosis by macrophages. J Exp Med.

[pbio-0060051-b017] Swanson JA, Johnson MT, Beningo K, Post P, Mooseker M (1999). A contractile activity that closes phagosomes in macrophages. J Cell Sci.

[pbio-0060051-b018] Araki N, Hatae T, Furukawa A, Swanson JA (2003). Phosphoinositide-3-kinase-independent contractile activities associated with Fcgamma-receptor-mediated phagocytosis and macropinocytosis in macrophages. J Cell Sci.

[pbio-0060051-b019] Olazabal IM, Caron E, May RC, Schilling K, Knecht DA (2002). Rho-kinase and myosin-II control phagocytic cup formation during CR, but not FcgammaR, phagocytosis. Curr Biol.

[pbio-0060051-b020] Cox D, Berg JS, Cammer M, Chinegwundoh JO, Dale BM (2002). Myosin X is a downstream effector of PI(3)K during phagocytosis. Nat Cell Biol.

[pbio-0060051-b021] Tuxworth RI, Weber I, Wessels D, Addicks GC, Soll DR (2001). A role for myosin VII in dynamic cell adhesion. Curr Biol.

[pbio-0060051-b022] Guminska M, Ptak W, Zembala M (1975). Macrophage metabolism during phagocytosis and digestion of normal and IgG antibody-coated sheep erythrocytes. Enzyme.

[pbio-0060051-b023] Loike JD, Kozler VF, Silverstein SC (1979). Increased ATP and creatine phosphate turnover in phagocytosing mouse peritoneal macrophages. J Biol Chem.

[pbio-0060051-b024] Wyss M, Kaddurah-Daouk R (2000). Creatine and creatinine metabolism. Physiol Rev.

[pbio-0060051-b025] Dzeja PP, Terzic A (2003). Phosphotransfer networks and cellular energetics. J Exp Biol.

[pbio-0060051-b026] Wallimann T, Wyss M, Brdiczka D, Nicolay K, Eppenberger HM (1992). Intracellular compartmentation, structure and function of creatine kinase isoenzymes in tissues with high and fluctuating energy demands: the ‘phosphocreatine circuit' for cellular energy homeostasis. Biochem J.

[pbio-0060051-b027] van Deursen J, Heerschap A, Oerlemans F, Ruitenbeek W, Jap P (1993). Skeletal muscles of mice deficient in muscle creatine kinase lack burst activity. Cell.

[pbio-0060051-b028] Jost CR, Van Der Zee CE, In ‘t Zandt HJ, Oerlemans F, Verheij M (2002). Creatine kinase B-driven energy transfer in the brain is important for habituation and spatial learning behaviour, mossy fibre field size and determination of seizure susceptibility. Eur J Neurosci.

[pbio-0060051-b029] Bernstein BW, Bamburg JR (2003). Actin-ATP hydrolysis is a major energy drain for neurons. J Neurosci.

[pbio-0060051-b030] Kreutzberg GW (1996). Microglia: a sensor for pathological events in the CNS. Trends Neurosci.

[pbio-0060051-b031] Loike JD, Kozler VF, Silverstein SC (1984). Creatine kinase expression and creatine phosphate accumulation are developmentally regulated during differentiation of mouse and human monocytes. J Exp Med.

[pbio-0060051-b032] Castellano F, Le Clainche C, Patin D, Carlier MF, Chavrier P (2001). A WASp-VASP complex regulates actin polymerization at the plasma membrane. EMBO J.

[pbio-0060051-b033] Koretsky AP, Traxler BA (1989). The B isozyme of creatine kinase is active as a fusion protein in Escherichia coli: in vivo detection by 31P NMR. FEBS Lett.

[pbio-0060051-b034] Hornemann T, Rutishauser D, Wallimann T (2000). Why is creatine kinase a dimer? Evidence for cooperativity between the two subunits. Biochim Biophys Acta.

[pbio-0060051-b035] Allen LA, Aderem A (1996). Molecular definition of distinct cytoskeletal structures involved in complement- and Fc receptor-mediated phagocytosis in macrophages. J Exp Med.

[pbio-0060051-b036] Beningo KA, Wang YL (2002). Fc-receptor-mediated phagocytosis is regulated by mechanical properties of the target. J Cell Sci.

[pbio-0060051-b037] Champion JA, Mitragotri S (2006). Role of target geometry in phagocytosis. Proc Natl Acad Sci U S A.

[pbio-0060051-b038] Lim J, Wiedemann A, Tzircotis G, Monkley SJ, Critchley DR (2007). An essential role for talin during alpha(M)beta(2)-mediated phagocytosis. Mol Biol Cell.

[pbio-0060051-b039] Galbraith CG, Yamada KM, Galbraith JA (2007). Polymerizing actin fibers position integrins primed to probe for adhesion sites. Science.

[pbio-0060051-b040] Kress H, Stelzer EH, Holzer D, Buss F, Griffiths G (2007). Filopodia act as phagocytic tentacles and pull with discrete steps and a load-dependent velocity. Proc Natl Acad Sci U S A.

[pbio-0060051-b041] Pantaloni D, Le Clainche C, Carlier MF (2001). Mechanism of actin-based motility. Science.

[pbio-0060051-b042] Loisel TP, Boujemaa R, Pantaloni D, Carlier MF (1999). Reconstitution of actin-based motility of Listeria and Shigella using pure proteins. Nature.

[pbio-0060051-b043] Steeghs K, Benders A, Oerlemans F, de Haan A, Heerschap A (1997). Altered Ca2+ responses in muscles with combined mitochondrial and cytosolic creatine kinase deficiencies. Cell.

[pbio-0060051-b044] Kraft T, Hornemann T, Stolz M, Nier V, Wallimann T (2000). Coupling of creatine kinase to glycolytic enzymes at the sarcomeric I-band of skeletal muscle: a biochemical study in situ. J Muscle Res Cell Motil.

[pbio-0060051-b045] Campanella ME, Chu H, Low PS (2005). Assembly and regulation of a glycolytic enzyme complex on the human erythrocyte membrane. Proc Natl Acad Sci U S A.

[pbio-0060051-b046] Garin J, Diez R, Kieffer S, Dermine JF, Duclos S (2001). The phagosome proteome: insight into phagosome functions. J Cell Biol.

[pbio-0060051-b047] Hornemann T, Kempa S, Himmel M, Hayess K, Furst DO (2003). Muscle-type creatine kinase interacts with central domains of the M-band proteins myomesin and M-protein. J Mol Biol.

[pbio-0060051-b048] Chida K, Kasahara K, Tsunenaga M, Kohno Y, Yamada S (1990). Purification and identification of creatine phosphokinase B as a substrate of protein kinase C in mouse skin in vivo. Biochem Biophys Res Commun.

[pbio-0060051-b049] Aksenov M, Aksenova M, Butterfield DA, Markesbery WR (2000). Oxidative modification of creatine kinase BB in Alzheimer's disease brain. J Neurochem.

[pbio-0060051-b050] Iwabata H, Yoshida M, Komatsu Y (2005). Proteomic analysis of organ-specific post-translational lysine-acetylation and -methylation in mice by use of anti-acetyllysine and -methyllysine mouse monoclonal antibodies. Proteomics.

[pbio-0060051-b051] Zhao TJ, Yan YB, Liu Y, Zhou HM (2007). The generation of the oxidized form of creatine kinase is a negative regulation on muscle creatine kinase. J Biol Chem.

[pbio-0060051-b052] Zurmanova J, Difato F, Malacova D, Mejsnar J, Stefl B (2007). Creatine kinase binds more firmly to the M-band of rabbit skeletal muscle myofibrils in the presence of its substrates. Mol Cell Biochem.

[pbio-0060051-b053] Lee WL, Bezanilla M, Pollard TD (2000). Fission yeast myosin-I, Myo1p, stimulates actin assembly by Arp2/3 complex and shares functions with WASp. J Cell Biol.

[pbio-0060051-b054] Evangelista M, Klebl BM, Tong AH, Webb BA, Leeuw T (2000). A role for myosin-I in actin assembly through interactions with Vrp1p, Bee1p, and the Arp2/3 complex. J Cell Biol.

[pbio-0060051-b055] Colucci-Guyon E, Niedergang F, Wallar BJ, Peng J, Alberts AS (2005). A role for mammalian diaphanous-related formins in complement receptor (CR3)-mediated phagocytosis in macrophages. Curr Biol.

[pbio-0060051-b056] Romero S, Le Clainche C, Didry D, Egile C, Pantaloni D (2004). Formin is a processive motor that requires profilin to accelerate actin assembly and associated ATP hydrolysis. Cell.

[pbio-0060051-b057] Lee JH, Koh H, Kim M, Kim Y, Lee SY (2007). Energy-dependent regulation of cell structure by AMP-activated protein kinase. Nature.

[pbio-0060051-b058] Mahajan VB, Pai KS, Lau A, Cunningham DD (2000). Creatine kinase, an ATP-generating enzyme, is required for thrombin receptor signaling to the cytoskeleton. Proc Natl Acad Sci U S A.

[pbio-0060051-b059] Zhang H, Berg JS, Li Z, Wang Y, Lang P (2004). Myosin-X provides a motor-based link between integrins and the cytoskeleton. Nat Cell Biol.

[pbio-0060051-b060] Le Cabec V, Carreno S, Moisand A, Bordier C, Maridonneau-Parini I (2002). Complement receptor 3 (CD11b/CD18) mediates type I and type II phagocytosis during nonopsonic and opsonic phagocytosis, respectively. J Immunol.

[pbio-0060051-b061] Xia Y, Vetvicka V, Yan J, Hanikyrova M, Mayadas T (1999). The beta-glucan-binding lectin site of mouse CR3 (CD11b/CD18) and its function in generating a primed state of the receptor that mediates cytotoxic activation in response to iC3b-opsonized target cells. J Immunol.

[pbio-0060051-b062] Thornton BP, Vetvicka V, Pitman M, Goldman RC, Ross GD (1996). Analysis of the sugar specificity and molecular location of the beta-glucan-binding lectin site of complement receptor type 3 (CD11b/CD18). J Immunol.

[pbio-0060051-b063] Brown GD, Taylor PR, Reid DM, Willment JA, Williams DL (2002). Dectin-1 is a major beta-glucan receptor on macrophages. J Exp Med.

[pbio-0060051-b064] Hoppe AD, Swanson JA (2004). Cdc42, Rac1, and Rac2 display distinct patterns of activation during phagocytosis. Mol Biol Cell.

[pbio-0060051-b065] Carlisle HJ, Kennedy MB (2005). Spine architecture and synaptic plasticity. Trends Neurosci.

[pbio-0060051-b066] Hassan NF, Rifat S, Campbell DE, McCawley LJ, Douglas SD (1991). Isolation and flow cytometric characterization of newborn mouse brain-derived microglia maintained in vitro. J Leukoc Biol.

[pbio-0060051-b067] Michiels F, van der Kammen RA, Janssen L, Nolan G, Collard JG (2000). Expression of Rho GTPases using retroviral vectors. Methods Enzymol.

[pbio-0060051-b068] Hed J, Stendahl O (1982). Differences in the ingestion mechanisms of IgG and C3b particles in phagocytosis by neutrophils. Immunology.

[pbio-0060051-b069] Sistermans EA, de Kok YJ, Peters W, Ginsel LA, Jap PH (1995). Tissue- and cell-specific distribution of creatine kinase B: a new and highly specific monoclonal antibody for use in immunohistochemistry. Cell Tissue Res.

