# Supplementary material for: Creatine Kinase–Mediated ATP Supply Fuels Actin-Based Events in Phagocytosis
Source: PLoS Biol. 2008 Mar 11;6(3):e51. doi: 10.1371/journal.pbio.0060051 (PMC2265766; doi:10.1371/journal.pbio.0060051)
Supplement: Supplementary file 1 [file pbio.0060051.sd001.doc]

#### PROTOCOL S1 Kuiper *et al.*

### Supplemental Experimental Procedures

# Plasmid and retroviral expression vectors

CK-B cDNA was amplified by PCR from DNA from mouse clone EST AW551905 using a complementary 5’ primer 5’-TATACTCGAGCCATGCCCTTCTCCACCAGGC-3’ containing the start-codon of CK-B and a XhoI restriction site. The 3’ primer was a 20-mer poly-dT primer, also containing a XhoI restriction site. The obtained PCR product (the entire CK-B ORF and 3’ UTR) was subsequently cloned in XhoI-opened pEYFP-C1 (Clontech). For construction of pSG8puro-CK-B, the CK-B ORF was amplified by PCR from EST AA102913 using

5’-GCGGAATTCATGCCCTTCTCCAACAGC-3’ and 5’-CGCCTCGAGTCACTTCTGGGCCGGC-3’ as primers. The ORF was then cloned into the EcoRI and XhoI sites of pSG8puro [1]. Expression vector pSG8puro-ECFP-CK-B was constructed by first subcloning the CK-B open reading frame in (EcoRI/SalI) pECFP-C2 (Clontech) after which the ECFP-CK-B fusion was excised with NheI and DraI and subsequently blunt-ended with T4 DNA polymerase. The recipient vector pSG8puro was cut with EcoRI and XhoI, blunt-ended with T4 DNA polymerase and 5’-terminal phosphate groups were removed by shrimp alkaline phosphatase (Roche) according to the manufacturer’s protocols. The pSG8puro-ECFP control plasmid was generated by excising the CK-B ORF from pSG8puro-ECFP-CK-B with SacI and KpnI, followed by filling in the ends of the vector with T4 polymerase and subsequent religation of the vector.

To generate a vector for expression of catalytically inactive CK-B, cysteine-283 was replaced by a serine using the site-directed mutagenesis kit and protocol (QuikChange Site-Directed Mutagenesis kit, Stratagene) with the oligos 5’-CTACATCCTCACAAGCCCATCCAACCTG-3’ and 5’-CAGGTTGGATGGGCTTGTGAGGATGTAG-3’ and pSG8puro-CK-B or pECFP-C2-CK-B as template DNA. The sequences of all constructs were verified by sequencing. Retroviral expression constructs were created by insertion of the ORF’s of CK-B, CK-B_(C283S)_ and EYFP into retroviral vector DNA pLZRS-IRES-zeo [2], giving rise to pLZRS-CK-B, pLZRS-CK-B_(C283S)_ and pLZRS-EYFP, respectively.

# Transfection and retroviral transduction

Transient transfections of RAW 264.7 cells with pEYFP-based plasmid DNAs were performed using Lipofectamine 2000 (Invitrogen) according to manufacturer’s protocol. RAW 264.7 cells stably expressing EGFP-tagged ß-actin were generated by transfecting RAW 264.7 cells with pCMV-EGFP-β-actin, followed by neomycine selection. A population of cells with intermediate expression levels was obtained by FACS selection. To generate cells co-expressing EGFP-actin and ECFP-tagged CK-B or CK-B_(C283S)_, pSG8puro constructs were linearized with restriction enzyme PvuI and transfected into EGFP-actin RAW 264.7 cells using Lipofectamine 2000. Clones with stably integrated vector DNA were selected by the ability to grow in medium with puromycine (5 μg/ml).

RAW 264.7 cell lines expressing wildtype murine CK-B, catalytically inactive CK-B_(C283S)_ or EYFP were created by retroviral transduction, following procedures as described [2]. In short, pLZRS retroviral vector constructs were transfected into the ecotrophic packaging cell line, Phoenix ΦNX-A [2,3] using Lipofectamine 2000 (Invitrogen). Viral supernatants (2 ml from 35 mm dish) were harvested after 48 hours and added to RAW 264.7 cells grown in 35 mm dishes (Costar). After 24 hours the virus containing medium was replaced and after another 24 hours selection for stably transduced cells was initiated by adding Zeocin (0.5 mg/ml).

**Phagocytic targets and phagocytosis uptake assay**

Unlabeled zymosan particles (dead yeast particles; Sigma) were suspended in PBS and briefly dispersed by sonification. For fluorescent labeling, zymosan particles (10 mg) were incubated for 2 hours at room temperature in sodium carbonate buffer (0.1 M, pH 9.6) with fluorescein isothiocyanate (FITC) or tetramethylrhodamine isothiocyanate (TRITC) at a final concentration of 0.5 mg/ml. After extensive washing the labeled zymosan was collected by centrifugation, resuspended into single particles by sonification in PBS and stored in aliquots at -80°C. Complement opsonization was performed as described [4]. Cells were activated with 200 nM phorbol 12-myristate 13-acetate (PMA) 15 minutes prior to phagocytosis of complement-opsonized zymosan (COZ). IgG opsonized zymosan was prepared using zymosan A BioParticles® opsonizing reagent from Invitrogen, according to manufacturer’s protocol.

IgG coated 3-µm fluorescent polystyrene beads (Polysciences) were prepared as described [5]. Complement coated polystyrene beads were prepared in a similar fashion. Briefly, carboxylated 3-µm fluorescent polystyrene beads (Fluoresbrite® Yellow Green Carboxylate Microspheres, Polysciences) were incubated with mouse IgM (Sigma) in MES buffer (pH 6.7). After incubation with EDAC (Sigma) beads were washed three times with PBS, freshly isolated mouse serum was added and beads were incubated for 30 min at 37°C. After washing with 1% Triton X-100 in 10 mM Tris pH 9.4 and PBS, the beads were stored at 4°C in PBS with 0.03% fish skin gelatin and 2 mM sodium azide. Before use, beads were washed three times with PBS to remove the azide.

To determine phagocytic uptake efficiency, fluorescently labeled zymosan or coated polystyrene beads suspended in RPMI without phenol red were added to 1x10^5^ RAW 264.7 cells at a ratio of 10 particles per cell. After incubating 30 minutes at 37°C, the cells were washed 3 times with PBS. Subsequently, the cells were incubated with lyticase (100U/ml, Sigma) in PBS for 10 minutes at room temperature to remove extracellular zymosan. After rigorous trypsinization, the cells were diluted in RPMI containing 10% FCS and spun down. Cell pellets were resuspended in PBS + 1% paraformaldehyde and samples were microscopically analyzed to confirm removal of extracelullar particles. For quantification of phagocytosis, 1x10^4^ cells per sample were analyzed on a Becton-Dickinson FACScan flow cytometer. Mean fluorescence was calculated for 3 to 4 experiments performed in duplo.

**Fluorescence detection of CK-B and actin**

Cells grown on glass coverslips were fixed with 2% paraformaldehyde in PHEM buffer (25 mM HEPES, 10 mM EGTA, 60 mM PIPES, 2 mM MgCl_2_, pH 6.9), permeabilized with 0.1% Triton X-100 and incubated 20 min in PBS containing 4% bovine serum albumine (BSA). Primary antibodies used were polyclonal anti-CKB (1:2000) and monoclonal anti-CKB 21E10 (1:2000). Primary antibodies were detected by goat-anti-rabbit IgG or goat-anti-mouse IgG conjugated to Alexa Fluor 488 or Alexa Fluor 568 (Molecular Probes). Images were taken sequentially for each wavelength with a Biorad MRC1024 confocal microscope using an oil immersion 60x, 1.4 NA objective using the Lasersharp software (Biorad, version 4.2). In the case of saponin-extraction, cells were permeabilized with 0.05 % saponin in PBS for 1 minute prior to fixation to remove cytosolic protein.

**Live cell imaging**

Stably transfected RAW 264.7 cells expressing EYFP-CK-B or EYFP were cultured for 24 hours on glass bottomed Willco dishes (GWSt-3522) prior to live imaging. 30 minutes before each experiment, the culture medium was replaced by RPMI without phenol red (Gibco) containing 10 mM HEPES and 10% FCS. For dual imaging, RAW 264.7 cells stably expressing GFP-actin and ECFP constructs were cultured as described for the single transfectants.

A Zeiss LSM510meta confocal laser-scanning microscope was used, equipped with a temperature controlled CO_2_ incubator (type S) and sample stage. For recording we used a PlanApochromatic 63x, 1.4 NA oil immersion DIC lens (Carl Zeiss GmbH, Jena, Germany) to obtain optical slices of approximately 2.5 µm. To obtain the EGFP and ECFP signals, spectral recordings were taken with the meta-detector and separated using the linear unmixing option embedded in the Zeiss laser scanning microscope software (LSM 510 META version 3.2 SP2). Image acquisition was started after addition of zymosan particles.

For analysis of double transfected cells, regions of interest (ROIs) were placed around phagocytic cups and a portion of the cytosol next to the nucleus (also with embedded software). Accumulation of fluorescence in the phagocytic cup was calculated from the ratio between cup and cytosol. Alternatively, line plots of still images at the peak of accumulation were generated with ImageJ (National Institutes of Health, Bethesda, MD) software to determine accumulation of fluorescence in phagocytic cups of cells containing only EGFP-tagged constructs.

**Immunoblotting**

Cells were scraped on ice in lysis buffer (12.5 mM Na_2_HPO_4_, 2.8 mM KH_2_PO_4_, 0.05% Triton X-100, 0.3 mM DTT) containing protease inhibitor cocktail (Roche) and incubated on ice for 20 min. Cell lysates were separated on a 10% SDS-PAGE gel, and electrotransferred to a nitrocellulose membrane. Subsequently, membranes were blocked with PBS containing 5% skim milk, and incubated with anti-CK-B (21E10, diluted 1:2000) for 1 hr. Secondary horse radish peroxidase (HRP) conjugated goat-anti-mouse IgG (diluted 1:10^4^, Jackson Immunoresearch) was used in combination with the HRP substrate Lumi-Light (Roche) for detection of protein bands.

**CK-activity assay**

CK-activity was determined by enzyme-coupled reactions. Briefly, cell lysates were prepared as described (see immunoblotting) and protein content was determined. Lysates with equal protein content were serially diluted in PBS in a 96-wells plate (Corning). Then 100 μl assay mix, consisting of bis-Tris acetate buffer (0.1 M, pH 6.9), hexokinase (1 u/ml), **glucose-6-phosphate dehydrogenase (0.8 u/ml), phosphocreatine (15 mM), glucose (2 mM), ADP (2 mM), NADP (2 mM), MgCl_2_ (10 mM) and** 10 μm bis(adenosine-5')-pentaphosphate (Ap5A), **was added. Upon addition of 10% vol/vol Wst-1 reagent (Roche), CK-mediated formazan generation was measured every minute on a BioRad 3550 micro-plate reader at 450 nm. CK-activity was expressed in arbitrary units.**

**Adhesion assay**

Cells were cultured on coverslips in 24-wells plates (1x10^5^ per well) overnight. In some experiments cells were pre-treated (30 min) with 50 and 100 nM Cytochalasin D (Sigma). After PMA stimulation (200 nM, 15 min) and three subsequent PBS washes with serum-free RPMI, FITC-labeled COZ particles were added (10 per cell) to the cells for 30 minutes at 37°C. Cells were washed 2 times with PBS to remove non-bound particles upon fixation (30 minutes at room temperature) with PHEM buffer containing 2% paraformaldehyde and 0.5% glutaraldehyde. Subsequently, the coverslips were washed with PBS, incubated in PBS/glycine/BSA for 20 minutes and external zymosan was stained with anti-zymosan IgG (1:500, Molecular Probes) followed by permeabilization (3 minutes 0.1% Triton X-100 in PBS) and incubation with secondary Alexa 660 conjugated goat-anti-rabbit IgG (1:300, Molecular Probes). Cells were counterstained with Alexa 568 conjugated phalloidin. In three independent experiments 9 random fields were imaged for each cell line with a Biorad MRC1024 confocal microscope using an oil immersion 60x objective. The total number of particles and the number of external adherent particles per cell were calculated.

For assessment of receptor-blocking effects, coverslips with cells were placed on parafilm (cells facing up) and incubations took place with smaller volumes. Involvement of CR3 was tested by pre-treating cells with either 100 μl RPMI containing 20 μg/ml human IgG (control) or M1/70 (anti-CR3; culture-supernatant, 1:25). COZ and IgG-opsonized zymosan were incubated for 30 minutes at 37°C in 200 μl RPMI containing either human IgG (20 μg/ml) or M1/70. Cells were subsequently washed and fixed as described. The number of particles (bound and internalized) was determined and normalized to the control. To block Fc-γRs human IgG (3 mg/ml) was added to the coverslips (30 min pre-incubation and during adhesion). BSA (3 mg/ml) served as control.

# F-actin quantification

F-actin quantification was performed essentially as described [6]. Briefly, RAW 264.7 cells were grown on non-tissue culture treated petri-dishes and incubated for 2 hours in presence or absence of 5 mM cyclocreatine prior to harvesting by scraping in PBS containing BSA (1%) and EDTA (5 mM). Subsequently, cells were washed once with RPMI and fixed with 4% paraformaldehyde in PHEM buffer. Small aliquots were permeabilized (PBS containing 0.1 % Triton X-100 and 2.5% BSA) and stained with Alexa 660 conjugated to phalloidin (Molecular Probes). Mean fluorescence (Fl-4) was determined by FACS (FacsCalibur, Becton Dickinson).

# FRAP experiment

To study the relative mobilities of CK-B and actin in the phagocytotic cup we stably transfected RAW264.7 cells with ECFP-CK-B and EYFP-ß-actin. CFP and YFP images were collected sequentially. CFP was excited at 458 nm at moderate laser power, and emission was detected using a 470–500 nm bandpass filter and YFP was excited at 514 nm at moderate laser power, and emission was detected using a 560-nm longpass filter. After 10 images CFP and YFP fluorescence in the phagocytic cup were bleached by scanning a square region covering approximately half the cup area 20 times at high laser power. After photobleaching, recovery was monitored for 30 sec. The recovery curves were normalized by calculating *I*_norm_ = (*I* – *I*_bg_)/(*I*_pre_ – *I*_bg_), where *I*_pre_, is the fluorescent intensity before the bleach and *I*_bg_ the background intensity, and the T1/2 of recovery was determined.

**REFERENCES**

1. de Bruin W, Oerlemans F, Wieringa B (2004) Adenylate kinase I does not affect cellular growth characteristics under normal and metabolic stress conditions. Exp Cell Res 297: 97-107.

2. Michiels F, van der Kammen RA, Janssen L, Nolan G, Collard JG (2000) Expression of Rho GTPases using retroviral vectors. Methods Enzymol 325: 295-302.

3. Kinsella TM, Nolan GP (1996) Episomal vectors rapidly and stably produce high-titer recombinant retrovirus. Hum Gene Ther 7: 1405-1413.

4. Hed J, Stendahl O (1982) Differences in the ingestion mechanisms of IgG and C3b particles in phagocytosis by neutrophils. Immunology 45: 727-736.

5. Kress H, Stelzer EH, Holzer D, Buss F, Griffiths G, et al. (2007) Filopodia act as phagocytic tentacles and pull with discrete steps and a load-dependent velocity. Proc Natl Acad Sci U S A 104: 11633-11638.

6. Geneste O, Copeland JW, Treisman R (2002) LIM kinase and Diaphanous cooperate to regulate serum response factor and actin dynamics. J Cell Biol 157: 831-838.
